# Real-world Effectiveness of the Adjuvanted Recombinant Zoster Vaccine in ≥50-year-old Adults With Autoimmune Diseases

**DOI:** 10.1093/infdis/jiaf395

**Published:** 2025-08-11

**Authors:** Dagna Constenla, Germain Lonnet, Emmanuel Aris, Ramsanjay Rk, Nathalie Servotte, Agnes Mwakingwe-Omari, Hannah Alsdurf, Huifeng Yun

**Affiliations:** GSK, Rockville, Maryland, USA; GSK, Wavre, Belgium; GSK, Wavre, Belgium; GSK, Bengaluru, Karnataka, India; GSK, Wavre, Belgium; GSK, Rockville, Maryland, USA; GSK, Rockville, Maryland, USA; GSK, Rockville, Maryland, USA

**Keywords:** herpes zoster, vaccine effectiveness, autoimmune diseases, herpes zoster vaccine, vaccination

## Abstract

**Background:**

Real-world data on the vaccine effectiveness (VE) of the adjuvanted recombinant zoster vaccine (RZV) to prevent herpes zoster (HZ) among individuals with autoimmune diseases (AIDs) are limited. To address this knowledge gap, we aimed to evaluate the VE of 2 RZV doses against HZ in ≥50-year-old adults with selected AIDs (rheumatoid arthritis, inflammatory bowel disease, systemic lupus erythematosus, multiple sclerosis (MS), psoriasis (PsO), and psoriatic arthritis).

**Methods:**

We conducted a retrospective matched cohort study using Optum's deidentified Clinformatics® Data Mart Database datasets from January 2018 to December 2021. Patients were matched by AID condition, age, and medication category, then 1:3 by propensity scores that accounted for the likelihood of receiving RZV, adjusted on selected confounders. For each AID, we calculated HZ incidence rates and RZV VE, overall and stratified by age, sex, time interval between 2 RZV doses, medication category, and time since vaccination.

**Results:**

The 2-dose cohort included 36 645 RZV-vaccinated and 109 229 unvaccinated patients. Two RZV doses offer protection against HZ in patients with AIDs, with VEs ranging from 48.1% for MS to 77.2% for PsO. An overall reduction in HZ incidence from 12.9 (95% confidence interval [CI]: 12.3; 13.5) to 4.3 (95% CI: 3.8; 4.9) per 1000 person-years among vaccinated individuals was found, corresponding to an overall VE against HZ of 66.3% (95% CI: 61.4; 70.7).

**Conclusions:**

Our analysis shows that RZV vaccination prevents HZ in ≥50-year-old adults with selected AIDs, consistent with prior studies.

## Teaser

Data on RZV's effectiveness against herpes zoster in patients with autoimmune diseases (AIDs) is limited. Vaccine effectiveness ranged from 48% for multiple sclerosis to 77% for psoriasis. Two RZV doses effectively prevent herpes zoster in ≥50-year-olds with selected AIDs.

## Graphical Abstract

**Graphical Abstract:**
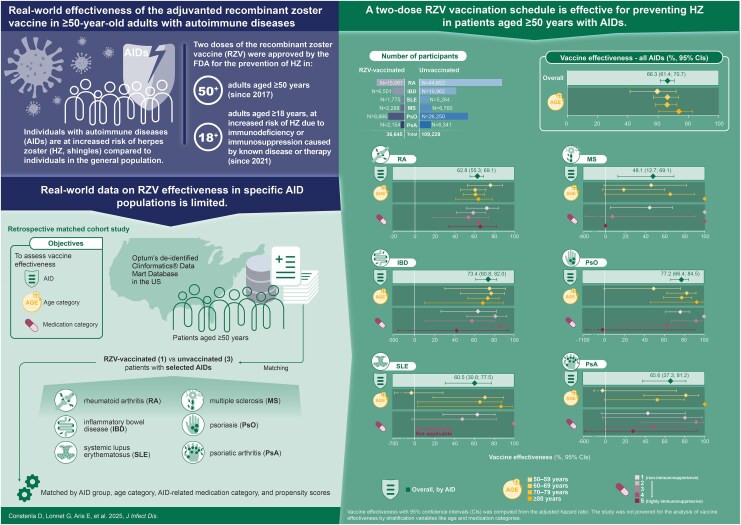

Herpes zoster (HZ) is a painful cutaneous eruption caused by the reactivation of the varicella-zoster virus. Before the introduction of HZ vaccines, it was estimated that almost one-third of United States (US) residents experienced HZ during their lifetime [1].

Individuals with autoimmune diseases (AIDs), such as rheumatoid arthritis (RA), inflammatory bowel disease (IBD), systemic lupus erythematosus (SLE), multiple sclerosis (MS), psoriasis (PsO), or psoriatic arthritis (PsA), and those undergoing AID-specific immunosuppressive therapy are at increased risk of developing HZ compared with the general population [2–10]. In 2017, the US Food and Drug Administration (FDA) approved, and the Advisory Committee on Immunization Practices (ACIP) recommended, 2 doses of the subunit zoster vaccine containing recombinant glycoprotein E in combination with the adjuvant AS01_B_ (RZV, *Shingrix*, GSK) for the prevention of HZ in ≥50-year-old adults [11, 12]. In 2021, the FDA approved 2 RZV doses in ≥18-year-old adults who are or will be at increased risk of HZ due to immunodeficiency or immunosuppression caused by known disease or therapy [13] and ACIP expanded their recommendations to include ≥19-year-old adults who are or will be immunodeficient or immunosuppressed because of disease or therapy [12].

In prelicensure studies (ZOE-50/70), RZV efficacy against HZ was ≥89.8% in ≥50-year-old adults [14, 15]. However, these studies did not include immunosuppressed individuals, who may benefit the most from HZ vaccination. A post-hoc analysis of data from ZOE-50/70 participants with pre-existing potential immune-mediated diseases at enrollment found that 2 RZV doses have an overall efficacy of 90.5% against HZ [16]. Subsequent studies evaluated vaccine efficacy and vaccine effectiveness (VE) in immunocompromised populations [17–19]. Yet, data on RZV VE for specific AIDs remain limited.

This real-world evidence study evaluated the 2-dose RZV VE against HZ in ≥50-year-old adults with selected AIDs.

## METHODS

### Study Design and Data Sources

We conducted a retrospective matched cohort study using datasets from Optum's deidentified Clinformatics® Data Mart Database (CDM) between January 2018 and December 2021. CDM is an administrative, retrospective database that includes proprietary, deidentified health claims data (eg, diagnoses, procedures, and pharmacy claims) for members of large commercial and Medicare Advantage health plans (Optum®; Optum, Inc, MN, US). Spanning all 50 states, it is representative of the US working population, their dependents, and the individuals enrolled under a Medicare supplemental health plan [20].

**Figure 1.**
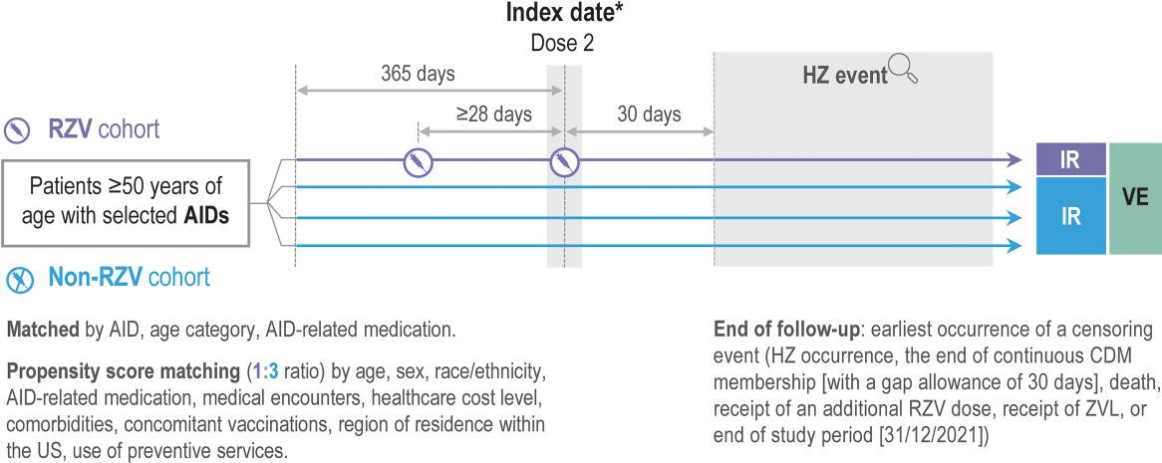
Study design. *The index date for the 1-dose and 2-dose cohorts was the date of receipt of the first and second RZV dose, respectively. The index date for unvaccinated patients corresponded to that of the vaccinated patients. Note: Propensity score matching was done at the index date. For each AID condition and sensitivity analyses, separate propensity scores and different sets of potential confounders were included. For stratified analysis with matched variables, individuals were matched on AID condition, age group, and medication category; then 1:3 matched on propensity scores with the list of covariates. Abbreviations: AID, autoimmune disease; CDM, Optum Clinformatics® Data Mart Database; HZ, herpes zoster; IR, incidence rate; non-RZV cohort, unvaccinated patients; RZV, adjuvanted recombinant zoster vaccine; RZV cohort, RZV-vaccinated patients; US, United States; VE, vaccine effectiveness; ZVL, live-attenuated varicella-zoster virus vaccine.

Age, immunodeficiency due to the disease itself, and immunosuppressive medication are considered the main risk factors for HZ [21–23] and, hence, could play a role as confounders in the effect of vaccination on the prevention of HZ. Individuals who received 2 RZV doses ≥28 days apart were matched with unvaccinated individuals within the same AID group, by age category (5-year increments), by AID-related medication category (medication categories 1–4 [based on their immunosuppressive intensity] were mutually exclusive, Supplementary Table 1), and in a 1:3 ratio by propensity scores that accounted for the likelihood of receiving RZV, adjusted on selected confounders (Figure 1). Propensity scores, defined as the probability of a patient receiving a particular treatment based on their observed characteristics, were calculated using logistic regression models with receipt of RZV as the dependent variable and age (5-year increments), sex, race/ethnicity, use of AID-related medications, medical encounters (number of inpatient admissions, ambulatory/emergency department/specialist outpatient visits), healthcare cost level, presence of comorbidities (kidney/cardiovascular/pulmonary/liver diseases, other AIDs, diabetes mellitus, cancer, immunocompromising conditions, and COVID-19 diagnosis), concomitant vaccinations during baseline period (influenza, tetanus-diphtheria-pertussis, and pneumococcal vaccines), region of residence within the US, and use of preventive services as independent variables.

The index date for patients in the 2-dose cohort was defined as the date of receipt of the second RZV dose; the same index date was assigned to both vaccinated (RZV cohort) and unvaccinated patients (non-RZV cohort) (Figure 1). A similar approach was used for patients receiving 1 RZV dose (one-dose cohort); the index date was defined as the date of receipt of RZV, and the same date was assigned to unvaccinated counterparts.

This study was conducted in accordance with the protocol and the ethical principles derived from international guidelines including the Declaration of Helsinki and Council for International Organizations of Medical Sciences International Ethical Guidelines, FDA's Code of Federal Regulations Title 21, and all other applicable regulations and local laws. Data privacy was protected by using anonymized data.

### Study Population

The study population included ≥50-year-old adults at index date who were diagnosed with 1 or more selected AIDs—RA, IBD (including ulcerative colitis [UC] and Crohn's disease [CD]), SLE, MS, PsO, PsA—and received the first RZV dose anytime between 1 January 2018 and 31 December 2021, and their unvaccinated matches. Full inclusion and exclusion criteria are available in Supplementary Table 2.

Individuals with selected AIDs in the CDM were identified in the year before the index date using modified versions of algorithms (Supplementary Table 3), which used data from the published literature defining AID conditions [24–31]. If patients met >1 AID definition, they were included in multiple cohorts, and AIDs were analyzed separately. RZV vaccination was identified from administrative claims using Current Procedural Terminology (code 90750) and National Drug Code (codes 58160-828-01/58160-829-01/58160-819-12/58160-828-03/58160-829-03/58160-823-11).

Patients were followed up from 30 days after the index date until the earliest occurrence of a censoring event (Figure 1). The main outcome was the HZ event, defined by the occurrence of either 1 inpatient claim with an HZ diagnosis (International Classification of Diseases, 10th Revision codes: B02.xx), or 2 outpatient claims with an HZ diagnosis ≤30 days apart, or 1 outpatient claim with HZ diagnosis with a pharmacy claim for antiviral treatment (acyclovir/valacyclovir/famciclovir) within 7 days before or after the HZ diagnosis claim.

If unvaccinated participants received a RZV dose, they were then included in the 1-dose cohort. Individuals in the 1-dose cohort were followed for 6 months or until their second RZV dose, if earlier. Individuals in the 1-dose cohort who received a second RZV dose <1 year apart and met the inclusion and exclusion criteria were included in the 2-dose cohort. Patients who received 2 RZV doses ≥1 year apart were considered outliers and removed from the 2-dose analysis.

### Objectives

The primary objective of this study was to estimate the overall VE against HZ after 2 RZV doses in ≥50-year-old patients with RA, IBD, SLE, MS, PsO, or PsA. Secondary objectives were to evaluate the VE against HZ after 2 RZV doses, stratified by age, sex, time interval between 2 RZV doses, AID-related medication category (mutually exclusive) based on current use at index date, and time since vaccination, and to evaluate the VE against HZ after 1 RZV dose.

### Statistical Methods

Patients’ baseline characteristics in the RZV and non-RZV cohorts were analyzed descriptively.

For the primary analyses, HZ incidence rates (IRs) were calculated by dividing the number of HZ cases by the total number of person-years. The number of person-years represents the sum of the duration of the risk periods (ie, 30 days postindex date until the earliest occurrence of a censoring event) of all patients included in the cohort. Adjusted hazard ratios (aHRs) and 95% confidence intervals (CIs) were obtained from Cox proportional hazards regression models. VE estimates and 95% CIs were computed from aHRs: VE = (1−aHR) × 100. Analyses for secondary objectives employed similar statistical methods as the primary analyses.

Sensitivity analyses were performed to assess the impact of the COVID-19 pandemic on the VE among patients in the 2-dose cohort who tested positive for SARS-CoV-2 during the follow-up period; the impact of including medication use as a covariate in the primary analysis; and the impact of overlaps between PsO and PsA diagnoses on the overall VE in 1- and 2-dose cohorts and on the stratified VE in the 2-dose cohort.

Since this study was not powered to interpret the secondary endpoints effectively, specific categories (age groups, time interval between 2 RZV doses, medication categories, and time since vaccination) were pooled in post-hoc analyses to provide additional statistical power and increase data interpretability. The study was not powered to run interaction testing, so no analyses on effect comparison were performed.

Analyses were conducted using SAS v9.4 (SAS Institute, NC, US).

## RESULTS

### Characteristics of the Patient Population

The RZV cohort included 36 645 patients and the non-RZV cohort included 109 229 matched patients. The RZV cohort comprised 15 061 patients with RA, 6501 patients with IBD, 1775 patients with SLE, 2288 patients with MS, 8866 patients with PsO, and 2154 patients with PsA (Table 1). In both RZV and non-RZV cohorts, the majority of RA and PsA patients received category 4 medications. For IBD, SLE, MS, and PsO, category 1 medications were most common in both cohorts (Table 1).

**Table 1.** Baseline characteristics of the patient population in the 2-dose cohort after matching by vaccination status

AID	All AIDs		RA		IBD		SLE		MS		PsO		PsA	
Cohort	RZV	Non-RZV	RZV	Non-RZV	RZV	Non-RZV	RZV	Non-RZV	RZV	Non-RZV	RZV	Non-RZV	RZV	Non-RZV
*N*	36 645	109 229	15 061	44 652	6501	19 962	1775	5264	2288	6760	8866	26 250	2154	6341
Females; %	65.0	64.9	73.3	73.4	54.7	54.7	88.9	89.2	75.7	75.2	53.2	53.2	54.5	53.9
Comorbidities^a^; %	58.6	58.6	63.1	63.0	56.5	56.9	66.0	65.0	44.4	44.4	54.1	54.2	61.3	61.0
Years of follow-up; mean	1.4	1.3	1.5	1.3	1.5	1.3	1.4	1.3	1.4	1.3	1.4	1.3	1.4	1.2
Years of follow-up; median (IQR)	1.3 (0.7; 2.1)	1.1 (0.6; 2.0)	1.4 (0.8; 2.2)	1.1 (0.6; 2.0)	1.3 (0.8; 2.2)	1.1 (0.6; 2.0)	1.2 (0.7; 2.1)	1.0 (0.5; 1.9)	1.1 (0.7; 2.1)	1.0 (0.6; 1.9)	1.3 (0.7; 2.1)	1.1 (0.6; 1.9)	1.2 (0.7; 2.0)	1.0 (0.6; 1.9)
Age group; %														
50–59 y	16.0	15.9	12.9	12.8	16.3	16.4	20.3	20.4	27.6	27.5	15.9	15.8	21.0	20.9
60–69 y	35.5	35.5	33.8	33.8	33.1	33.3	38.1	37.9	44.5	44.5	35.9	35.9	40.6	40.7
70–79 y	37.0	37.1	38.9	39.0	38.6	38.3	33.7	33.7	24.3	24.4	37.7	37.8	32.5	32.5
≥80 y	11.5	11.6	14.4	14.5	12.0	11.9	7.9	7.9	3.6	3.6	10.5	10.5	5.9	5.9
Medication category^b,c^; %														
1	41.4	41.2	21.1	21.1	31.2	30.6	76.9	77.1	87.1	87.2	68.2	68.2	26.0	26.1
2	19.3	19.3	29.5	29.4	27.8	27.6	22.3	22.4	2.1	2.1	1.5	1.5	10.8	10.7
3	12.2	12.3	9.1	9.2	15.8	16.2	0.8	0.5	3.5	3.4	16.1	16.2	25.1	25.3
4	25.3	25.4	36.6	36.6	24.9	25.4	NA	NA	7.1	7.1	13.9	13.9	35.1	35.2
5	1.9	1.8	3.7	3.7	0.3	0.2	NA	NA	0.2	0.2	0.3	0.2	3.1	2.7

Abbreviations: AID, autoimmune disease; IBD, inflammatory bowel disease; IQR, interquartile range; MS, multiple sclerosis; *N*, total number of patients; NA, not applicable; non-RZV cohort, unvaccinated patients; PsA, psoriatic arthritis; PsO, psoriasis; RA, rheumatoid arthritis; RZV, adjuvanted recombinant zoster vaccine; RZV cohort, RZV-vaccinated patients; SARS-CoV-2, severe acute respiratory syndrome coronavirus 2; SLE, systemic lupus erythematosus.

^a^Comorbidities were identified through International Classification of Diseases, 10th Revision (ICD-10) diagnosis codes. Following comorbidities were considered:

Chronic diseases: Kidney disease (ICD-10 codes: I12.0, I12.9, I13.1*, N03.2-N03.7, N05.2-N05.7, N18.*, N19.*, N25.0, Z49.0*-Z49.2*, Z94.0, Z99.2); cardiovascular disease (ICD-10 codes: I21.*, I22.*, I25.2, I09.9, I11.0, I13.0, I13.2, I25.5, I42.0, I42.5-I42.9, I43.*, I50.*, P29.0); pulmonary disease (ICD-10 codes: I27.8*, I27.9, J40.*-J47.*, J60.*-J67.*, J68.4, J70.1, J70.3); liver disease (ICD-10 codes: B18.*, I85.0*, I86.4, I98.2, K70.0, K70.1*-K70.4*, K70.9, K71.1*, K71.3, K71.4, K71.5*, K71.7, K72.1*, K72.9*, K73.*, K74.*, K76.0, K76.2-K76.7, K76.8*, K76.9, Z94.4); diabetes mellitus (ICD-10 codes: E10.*-E14.*).

AIDs: RA (ICD-10 codes: M05.*, M06.*); IBD (ICD-10 codes: K50.*, K51.*); SLE (ICD-10 codes: M32.1, M32.8, M32.9); MS (ICD-10 code: G35); PsO/PsA (ICD-10 codes: L40.*, L40.5*).

Immunosuppressant conditions: Lymphoma/leukemia (ICD-10 codes: C81.*C86.*, C88.*, C90.*-C96.*, D45, D46.*); congenital and other immunodeficiencies (ICD-10 codes: D61.09, D61.3, D61.82, D61.9, D70.0, D71, D80.0, D80.1, D80.5, D80.8, D81.*, D82.*, D83.*, D84.0, D84.1, D84.89, D84.9, D89.81*, D89.82, D89.9, E31.0, E70.330, G11.3, Q82.4, Q89.0*); asplenia/hyposplenia (ICD-10 codes: D57.00, D57.01, D57.02, D57.1, D57.2, D57.20, D57.21, D57.211, D57.212, D57.219, D57.4, D57.40, D57.41, D57.411, D57.412, D57.419, D57.8, D57.80, D57.81, D57.811, D57.812, D57.819, D73.0, Q89.01, Q89.09, Z90.81).

SARS-CoV-2 infection (ICD-10 code: U07.1) and pneumonia due to COVID-19 (ICD-10 code: J12.82).

^b^Medication categories are detailed in Supplementary Table 1.

^c^The cutoff for steroid use is 5 mg for ≥93% of the patients with RA (5824 [93.1%] vaccinated patients; 16 791 [99.3%] unvaccinated patients), IBD (1398 [98.2%] vaccinated patients; 3923 [99.4%] unvaccinated patients), or PsA (514 [94.3%] vaccinated patients; 1620 [95.5%] unvaccinated patients). Less than 7% of patients with RA (429 [6.9%] vaccinated patients; 112 [0.7%] unvaccinated patients), IBD (25 [1.8%] vaccinated patients; 22 [0.6%] unvaccinated patients), or PsA (31 [5.7%] vaccinated patients; 77 [4.5%] unvaccinated patients) were placed in the low-dose steroid group.

Across all selected AIDs, most patients were females (RZV cohort: 65.0%, non-RZV cohort: 64.9%), aged 70–79 years at index date (RZV cohort: 37.0%, non-RZV cohort: 37.1%), and had ≥1 comorbidity (RZV cohort: 58.6%, non-RZV cohort: 58.6%). In the 2-dose cohort, 70.1% of patients were monitored until the end of the observation period, while 29.9% were censored due to the occurrence of HZ, receipt of an additional HZ vaccine, or death. The median follow-up was 1.3 years (interquartile range: 0.7; 2.1) and 1.1 years (0.6; 2.0) for RZV and non-RZV cohorts, respectively (Table 1). The rate of missing data was <5%.

The 1-dose cohort included 44 865 RZV-vaccinated and 130 458 unvaccinated patients. This cohort comprised 19 016 patients with RA, 7461 patients with IBD, 2294 patients with SLE, 2752 patients with MS, 10 815 patients with PsO, and 2527 patients with PsA who were RZV-vaccinated. Baseline characteristics for the 1-dose cohort are shown in Supplementary Table 4.

Most patients in the 2-dose (95.8%) and 1-dose (95.6%) cohorts had a distinct AID diagnosis. Less than 5% of patients had concurrent AID diagnoses.

### Vaccine Effectiveness Analyses

**Figure 2.**
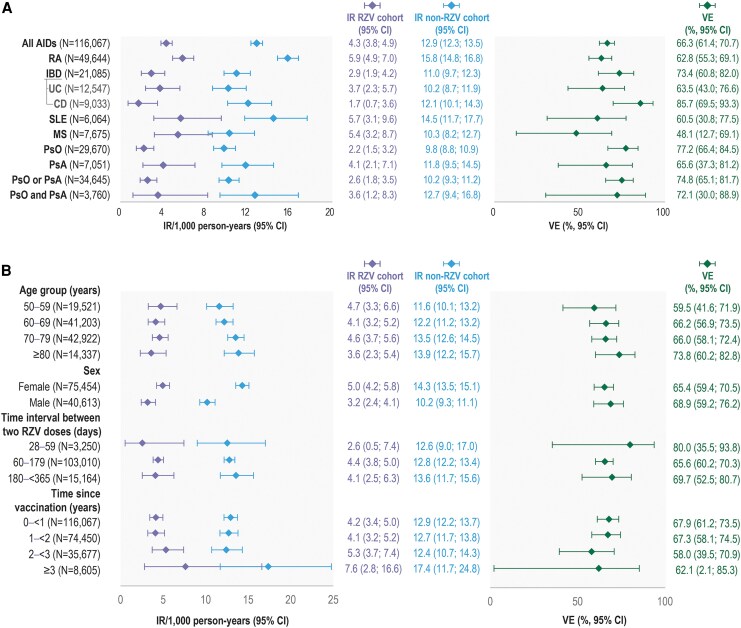
RZV VE against HZ after 2 RZV doses across selected AIDs. (*A*) Shows HZ IRs and RZV VE point estimates for each selected AID. (*B*) Shows HZ IRs and RZV VE point estimates for the selected AIDs altogether, stratified by age, sex, time interval between 2 RZV doses, and time since vaccination. Note: HZ IRs were calculated by dividing the number of HZ cases by the total number of person-years. aHRs and their 95% CIs were obtained from Cox proportional hazards regression models. The VE estimates and their 95% CIs were computed from aHRs: VE = (1−aHR) × 100. Abbreviations: AID, autoimmune disease; CD, Crohn's disease; CI, confidence interval; IBD, inflammatory bowel disease; IR, incidence rate; HZ, herpes zoster; MS, multiple sclerosis; *N*, number of patients; non-RZV cohort, unvaccinated patients; PsA, psoriatic arthritis; PsO, psoriasis; RA, rheumatoid arthritis; RZV, adjuvanted recombinant zoster vaccine; RZV cohort, RZV-vaccinated patients; SLE, systemic lupus erythematosus; UC, ulcerative colitis; VE, vaccine effectiveness.

The VE of 2 RZV doses against HZ were similar between AIDs (RA: 62.8% [95% CI: 55.3; 69.1], IBD: 73.4% [60.8; 82.0], SLE: 60.5% [30.8; 77.5], MS: 48.1% [12.7; 69.1], PsO: 77.2% [66.4; 84.5], and PsA: 65.6% [37.3; 81.2]; Figure 2*A*). The results for each of the selected AIDs stratified by age, sex, time interval between 2 RZV doses, medication use, and time since vaccination are shown in Supplementary Table 5. The results of the post-hoc analyses are shown in Supplementary Table 6.

HZ IRs for all selected AIDs were 4.3/1000 person-years (95% CI: 3.8; 4.9) in the RZV cohort and 12.9/1000 person-years (12.3; 13.5) in the non-RZV cohort, resulting in a VE of 66.3% (95% CI: 61.4; 70.7) (Figure 2*A*). When stratified by age, VE point estimates for all selected AIDs varied between 59.5% (95% CI: 41.6; 71.9) in the youngest age group (50–59 years) and 73.8% (60.2; 82.8) in the oldest age group (≥80 years). The VE was 68.9% (95% CI: 59.2; 76.2) in males and 65.4% (59.4; 70.5) in females. For the 60 to 179-day interval between 2 RZV doses (within the recommended interval of 2–6 months [32]), the VE was 65.6% (95% CI: 60.2; 70.3). VE point estimates varied by time elapsed since vaccination, but 95% CIs overlapped; VE was 62.1% (95% CI: 2.1; 85.3) ≥ 3 years postvaccination (Figure 2*B*).

In the 1-dose cohort, HZ IRs were 5.6/1000 person-years (95% CI: 4.3; 7.1) in RZV-vaccinated patients and 13.6/1000 person-years (12.7; 14.6) in unvaccinated patients. This resulted in a VE against HZ of 58.4% (95% CI: 45.9; 68.0) for all selected AIDs (Supplementary Figure 1).

### Sensitivity Analyses

When assessing the effect of SARS-CoV-2 infection on VE across all AID populations, outside of the pandemic-affected months (March 2020 to December 2020), there was no reduction in the number of HZ events or the total person-years of follow-up compared with the primary analysis. HZ IRs for all selected AIDs were 4.5/1000 person-years (95% CI: 3.9; 5.3) and 12.9/1000 person-years (12.2; 13.6) in the RZV and non-RZV cohorts, respectively. The sensitivity analysis yielded a VE against HZ of 64.8% (95% CI: 58.6; 70.0).

When evaluating the impact of medication use on the results of the primary analysis, the adjusted VEs of 2 RZV doses against HZ were 63.0% (95% CI: 55.5; 69.2) for RA, 73.5% (60.9; 82.0) for IBD, 60.6% (30.9; 77.5) for SLE, 48.1% (12.6; 69.1) for MS, 77.2% (66.5; 84.5) for PsO, 65.7% (37.5; 81.2) for PsA, and 66.5% (61.5; 70.8) for all selected AIDs.

When estimating the impact of overlaps between PsO or PsA diagnosis, the 2-dose VE against HZ was 74.8% (95% CI: 65.1; 81.7) in patients with PsO or PsA and 72.1% (30.0; 88.9) for patients with concurrent PsO and PsA diagnoses (Figure 2*A*); thus, the influence of PsA on the PsO analysis was minimal. VE estimates for the prevention of HZ after 2 RZV doses in patients with PsO and/or PsA, stratified by selected variables, are shown in Supplementary Table 5. Overall VE estimates against HZ for patients diagnosed with PsO and/or PsA who received 1 RZV dose are shown in Supplementary Figure 1.

## DISCUSSION

This study is one of the first to provide real-world data on the effectiveness of RZV in preventing HZ among ≥50-year-old patients with selected AIDs (RA/IBD/SLE/MS/PsO/PsA). The study assessed RZV VE against HZ for each AID, overall and stratified by age, sex, time interval between 2 RZV doses, medication categories, and time since vaccination. The results showed that 2 RZV doses offer adequate protection against HZ in patients with AIDs, with VEs ranging from 48.1% (MS) to 77.2% (PsO) and a VE of 66.3% (95% CI: 61.4; 70.7) across all AIDs.

The current study found that 2 RZV doses were 62.8% effective for patients with RA and 73.4% effective for patients with IBD. For patients with RA, a similar VE estimate (60.7%) was observed in a retrospective matched cohort study conducted among ≥50-year-old patients with the same diagnosis [33]. For IBD, another retrospective matched cohort study conducted among ≥50-year-old patients found a 2-dose VE against HZ of 65.1% [34]. Two RZV doses were previously shown to be 100% and 61.0% effective against HZ in 50–60-year-old and >60-year-old adults with IBD (including UC and CD), respectively [35].

The present study also showed that 2 RZV doses were 60.5% (95% CI: 30.8; 77.5) and 48.1% (95% CI: 12.7; 69.1) effective against HZ in patients with SLE or MS, respectively. A retrospective cohort study found a 2-dose RZV VE against HZ of 54% (95% CI: 18; 74) and 81% (95% CI: 70; 88) among commercially insured ≥18-year-old patients with SLE and MS, respectively [36]. Among Medicare-insured ≥18-year-old patients with SLE and MS, RZV's 2-dose VE against HZ was 70% (95% CI: 50; 82) and 64% (95% CI: 51; 74), respectively [36]. Although SLE data were consistent between both studies, in our study, 2 RZV doses were somewhat less effective against HZ in patients with MS. The somewhat lower 2-dose VE (and broad 95% CIs) against HZ in these patients can be attributed to the limited number of person-years data, a short follow-up period, and limited number of HZ events. Our study is one of the first to provide VE estimates for patients with PsO or PsA; future studies should focus on these specific AIDs to collect more comprehensive data and improve the accuracy of VE estimates.

Additionally, this study estimated RZV's 2-dose VE against HZ across selected AIDs, stratified by age, sex, time interval between 2 RZV doses, medication use, and time since vaccination. While VE data were generally interpretable for most AIDs, the assessments related to age and sex categories, dosing intervals, medication categories, and duration postvaccination were limited due to the small sample sizes in each group, and the short follow-up duration. To facilitate data interpretation for each AID, we conducted post-hoc analyses to recategorize relevant stratification variables, like age groups, time interval between 2 RZV doses, medication use, and time since vaccination. This approach provided more accurate estimates of VE for RA, IBD, and PsO, which had sufficient person-years of data from the initial subgroup analyses, as indicated by the narrower CIs. However, for other AIDs, merging categories did not enhance the accuracy of VE estimates for each factor.

Previously, the HZ IR among immunocompetent ≥50-year-old adults was estimated at 3.3/1000 person-years for those vaccinated with 2 RZV doses, compared to 10.6/1000 person-years for unvaccinated individuals [37]. Beyond the AID itself, immunosuppressive therapy considerably increases the risk of HZ [21, 23], particularly with the use of Janus kinase inhibitors [38]. In our study, we evaluated the VE in patients with AIDs, irrespective of whether they were on immunosuppressive medications; most patients were receiving treatments classified as category 1 (less immunosuppressive; for IBD, SLE, MS, or PsO) or category 4 (highly immunosuppressive; for RA or PsA) at index date. We observed an overall HZ IR of 4.3/1000 person-years in the RZV cohort and 12.9/1000 in the non-RZV cohort, corresponding to an overall VE against HZ of 66.3% (95% CI: 61.4; 70.7). These findings align with those from a cohort study involving ≥65-year-old Medicare Part D beneficiaries with AIDs, which reported HZ IRs of 4.4/1000 person-years for 2-dose RZV-vaccinated individuals and 14.9/1000 person-years for unvaccinated individuals and a 2-dose RZV VE of 68.0% (95% CI: 62.3; 72.8) [19]. However, the referenced study used a broader definition of AIDs (including 14 diseases) compared to our study, therefore, direct comparisons between these 2 studies should be interpreted with caution. A literature review and meta-analysis found a VE of 65% (95% CI: 57; 72) in immunocompromised adults (including, but not limited to, AIDs) vaccinated with RZV [39]. Our results suggest that both the underlying AID and AID-related medications contribute to an increased risk of HZ; however, this risk can be reduced through vaccination, as indicated by the lower IRs in the vaccinated versus non-vaccinated cohort.

RZV has demonstrated durable high efficacy (89.0%) up to at least 10 years postvaccination in ≥50-year-old adults [40]. While it is hypothesized that the duration of protection from RZV may be shorter for immunocompromised individuals, there is currently no published data to confirm this [23]. Our study is one of the first to show that 2 RZV doses are effective until ≥3 years postvaccination, with an overall VE of 62.1% across all AID populations. However, it is important to note that 95% CIs were quite broad for follow-up categories beyond 2 years.

Two RZV doses are recommended to ensure adequate protection against HZ [41]. Our findings indicate that a single dose provides a cumulative VE of 58.4% across selected AIDs. This VE is consistent with the previously estimated 57.7% VE for a 1-dose regimen among ≥65-year-old Medicare Part D community-dwelling beneficiaries with AIDs [19]. However, point estimates for 1-dose VE stratified by AID type were challenging to interpret due to broad and overlapping CIs for conditions such as SLE, IBD, PsO, or PsA. For MS, there were insufficient HZ events to compute a reliable VE.

This study has some limitations. First, the study was not statistically powered to effectively analyze the secondary endpoints. The low number of person-years and HZ events in the stratified analyses resulted in high variability in VE estimates, with overlapping CIs, complicating the interpretation of VE per category. To enhance the accuracy of VE estimates, we pooled relevant categories across stratification variables; the accuracy of VE estimates was enhanced for AIDs with sufficient person-years data available in the initial subgroup analysis. While our results should be interpreted with caution, they provide valuable insights for future studies, which should ensure adequate statistical power for stratified VE analysis based on specific medication categories and comorbid conditions to provide targeted vaccination recommendations for individuals with AIDs. Second, while the overall VE data categorized by age and sex were comprehensible, the assessments regarding dosing intervals and duration postvaccination were limited by insufficient person-year data. Additionally, owing to the retrospective nature of the study and the limitations inherent to the commercial claims database, there may have been confounding by indication, misclassification of exposures and outcomes, or selection bias, despite the adjustments we implemented. We addressed potential confounding using propensity score matching for all measured confounders and assessed the potential misclassification of low-dose steroids within the RA, IBD, and PsA groups. With <7% of patients with RA, IBD, or PsA placed in groups classified as receiving low-dose steroids, the risk of misclassifying high-potency steroids was minimal, which in turn did not notably impact the study's matching processes or conclusions. Furthermore, for most patients (70.1%), the follow-up period concluded by the study's end date, which reduced the likelihood of selection bias that often occurs when patients exit studies prematurely for outcome-related reasons. Additional analyses were conducted to address the inherent limitations of this observational study, and the rate of missing data for demographic variables was negligible (<5%).

Overall, our analyses provide real-world evidence that RZV effectively prevents HZ in ≥50-year-old patients with selected AIDs. Our findings are consistent with prior studies, demonstrating that RZV offers substantial benefits to this patient population. The results will inform both patient and physician decision-making regarding HZ vaccination and contribute to evidence-based recommendations and guidelines for vaccine uptake among at-risk populations. Nevertheless, further studies are needed to assess how various immunosuppressive medications and comorbidities affect RZV VE against HZ in AID populations. Additionally, research is warranted on RZV VE against HZ in younger populations with AIDs, particularly in light of the expanded FDA recommendations for immunocompromised ≥18-year-old individuals [41].

## Supplementary Material

jiaf395_Supplementary_Data

## Supplementary Data

Supplementary materials are available at *The Journal of Infectious Diseases* online (http://jid.oxfordjournals.org/). Supplementary materials consist of data provided by the author that are published to benefit the reader. The posted materials are not copyedited. The contents of all supplementary data are the sole responsibility of the authors. Questions or messages regarding errors should be addressed to the author.

## Notes

***Author contributions.*** D. C., G. L., E. A., A. M.-O., and H. Y. were involved in the conception or the design of the study. D. C., G. L., E. A., R. R., N. S., H. A., and H. Y. were involved in the analysis of the data. All authors were involved in data interpretation. All authors reviewed and commented on drafts of the manuscript for intellectual content and gave final approval to submit for publication. All authors attest they meet the ICMJE criteria for authorship.

***Acknowledgments.*** The authors thank Hao Wang for the contribution to this study. The authors also would like to thank the Akkodis Belgium platform for writing and editorial assistance (by Sara-Teodora Vulcu), manuscript coordination, and design support (by Ioana Sima and Ludivine van den Biggelaar) on behalf of GSK.

***Data availability.*** Please refer to GSK weblink to access GSK's data sharing policies and as applicable seek anonymized subject level data via the link https://www.gsk-studyregister.com/en/.

***Trademarks.***  *Shingrix* and AS01 are trademarks owned by or licensed to the GSK.

***Financial support.*** This work was supported by GSK, which was involved in all stages of the study conduct and analysis and paid for all costs associated with the development and publishing of this manuscript.

***Meeting(s) where the information has previously been presented.*** The results of this study were presented in-part in congress activities.

1. Constenla, D. et al. P986 Effectiveness of the adjuvanted recombinant zoster vaccine (RZV) in preventing herpes zoster (HZ) among patients ≥50 years of age with Inflammatory Bowel Disease in the United States: A retrospective real-world database analysis from 2018 to 2021. *J Crohns Colitis.*  **18,** i1779-80; 10.1093/ecco-jcc/jjad212.1116 (2024). Presented at: 19th Congress of European Crohn's and Colitis Organization (ECCO), February 21–24, 2024, Stockholm, Sweden.
2. Constenla, D. et al. POS1476-HPR THE ADJUVANTED RECOMBINANT ZOSTER VACCINE IS EFFECTIVE IN PATIENTS ≥50 YEARS OF AGE WITH RHEUMATOID ARTHRITIS AT INCREASED RISK OF HERPES ZOSTER: RESULTS FROM A REAL-WORLD DATABASE ANALYSIS IN THE UNITED STATES FROM 2018 TO 2021. *Ann Rheum Dis.*  **83,** 1189; 10.1136/annrheumdis-2024-eular.2950 (2024). Presented at: European Alliance of Associations for Rheumatology (EULAR) 2024, June 12–15, 2024, Vienna, Austria.
3. Constenla, D. et al. P-19 The Adjuvanted Recombinant Zoster Vaccine Is Effective in Preventing Herpes Zoster (HZ) Among Patients ≥50 Years of Age (YOA) With Selected Autoimmune Diseases in the United States: A Real-World Database Analysis (2018–2021). *Open Forum Infect Dis*. **12**, ofae631.227; 10.1093/ofid/ofae631.227 (2025). Presented at: IDWeek, October 16–19, 2024, Los Angeles, CA, US.
