# Supplementary material for: Real-world Effectiveness of the Adjuvanted Recombinant Zoster Vaccine in ≥50-year-old Adults With Autoimmune Diseases
Source: J Infect Dis. 2025 Aug 11;232(6):e931–40. doi: 10.1093/infdis/jiaf395 (PMC12718021; doi:10.1093/infdis/jiaf395)
Supplement: jiaf395_Supplementary_Data [file jiaf395_supplementary_data.docx]

**Real-world effectiveness of the adjuvanted recombinant zoster vaccine in ≥50-year-old adults with autoimmune diseases**

# Supplementary Information

## Supplementary Fig. 1: RZV VE against HZ after one RZV dose across selected AIDs


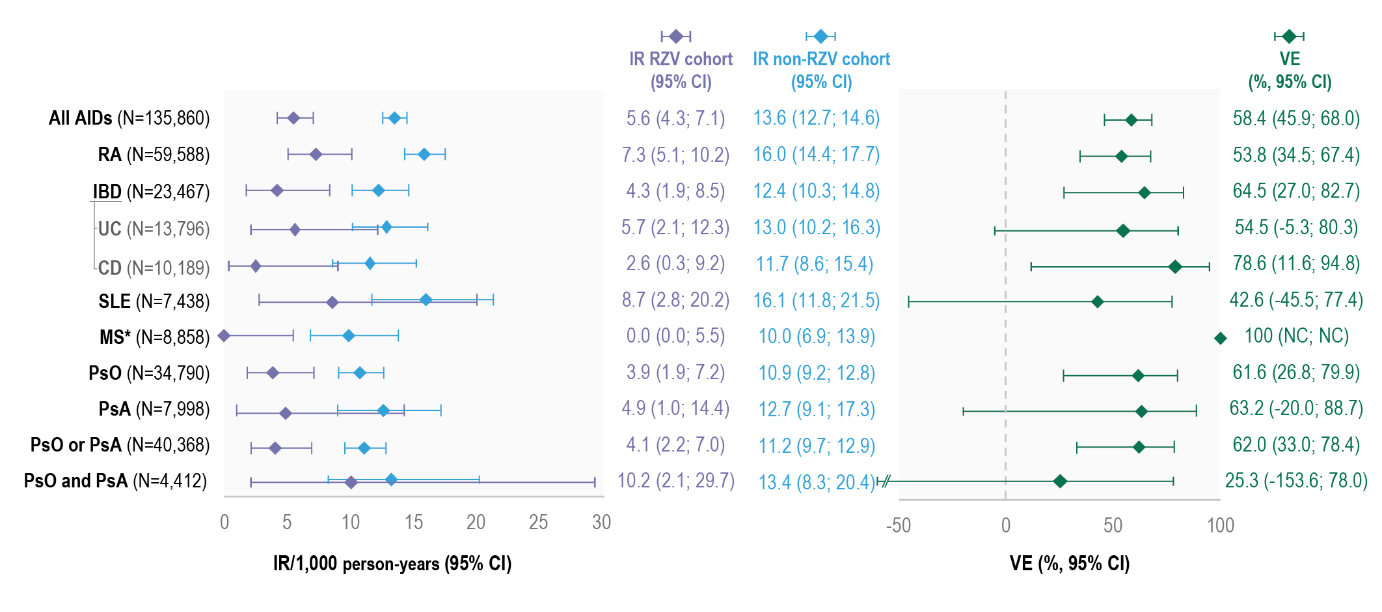


*The VE was not computable for patients with MS.

**Note:** HZ IRs were calculated by dividing the number of HZ cases by the total number of person-years. Adjusted hazard ratios (aHRs) and their 95% CIs were obtained from Cox proportional hazards regression models. The VE estimates and their 95% CIs were computed from aHRs: VE=(1−aHR)×100.

AID, autoimmune disease; CD, Crohn’s disease; CI, confidence interval; HZ, herpes zoster; IBD, inflammatory bowel disease; IR, incidence rate; MS, multiple sclerosis; N, total number of patients; NC, not computable; non-RZV cohort, unvaccinated patients; PsA, psoriatic arthritis; PsO, psoriasis; RA, rheumatoid arthritis; RZV, adjuvanted recombinant zoster vaccine; RZV cohort, RZV-vaccinated patients; SLE, systemic lupus erythematosus; UC, ulcerative colitis; VE, vaccine effectiveness.

## Supplementary Table 1: Medication categories for RA, IBD, SLE, MS, PsO, and PsA

| **Medication categories** | | **Notes** |
| --- | --- | --- |
| **RA medication categories** | | |
| Category 1 | No treatment  Or  NSAID (ibuprofen, naproxen, indomethacin, meloxicam, celecoxib, nabumetone, etodolac, diclofenac, sulindac, salsalate, ketorolac)  Or  Low-dose steroids (hydrocortisone, methylprednisolone, prednisolone, prednisone [all <5 mg or equivalent]) | Medication categories were determined by the highest category of medication prescribed during the search periods:  • Rituximab or rituximab-abbs/rituximab-pvvr (6 months before index date)  • Biologics (3 months before index date)  • JAK inhibitors (3 months before index date to 1 month after index date)  • NSAIDs, 5-ASA, aminosalicylate, sulfasalazine, thiopurines, steroids, and conventional DMARDs (medication prescribed/refilled within 6 months prior to index date)  • Any medication that is active at the index date was considered regardless of when it was prescribed/refilled. |
| Category 2 | Conventional DMARDs (hydroxychloroquine, leflunomide, MTX, sulfasalazine, minocycline, azathioprine) |  |
| Category 3 | Biologics (abatacept, rituximab, rituximab-abbs, rituximab-pvvr, rituximab-arrx, tocilizumab, sarilumab, adalimumab, etanercept, etanercept-szzs, etanercept-ykro, infliximab, infliximab-abda, infliximab-axxq, infliximab-dyyb, certolizumab pegol, golimumab, anakinra) |  |
| Category 4 | High-dose systemic steroids (any ≥5 mg prednisone or equivalent; hydrocortisone, methylprednisolone, prednisolone, prednisone) |  |
| Category 5 | JAK inhibitors (tofacitinib, baricitinib, upadicitinib) |  |
| **IBD medication categories** | | |
| Category 1 | No treatment | Medication categories were determined by the highest category of medication prescribed during the search periods:  • Rituximab or rituximab-abbs/rituximab-pvvr (6 months before index date)  • Biologics (3 months before index date)  • JAK inhibitors (3 months before index date to 1 month after index date)  • NSAIDs, 5-ASA, aminosalicylate, sulfasalazine, thiopurines, steroids, and conventional DMARDs (medication prescribed/refilled within 6 months prior to index date)  • Any medication that is active at the index date was considered regardless of when it was prescribed/refilled. |
| Category 2 | 5-ASA (5-ASA, sulfasalazine, olsalazine, mesalamine, balsalazide)  Or  Low-dose steroids (all <5 mg or equivalent; hydrocortisone, methylprednisolone, prednisolone, prednisone budesonide) |  |
| Category 3 | Biologics (infliximab, infliximab-abda, infliximab-axxq, infliximab-dyyb, adalimumab, adalimumab-adaz, adalimumab-adbm, adalimumab-atto, adalimumab-bwwd, vedolizumab, ustekinumab, golimumab, certolizumab, natalizumab)  Or  Conventional DMARD (MTX)  Or  Thiopurines (azathioprine, mercaptopurine, thioguanine) |  |
| Category 4 | High-dose systemic steroids (any ≥5 mg prednisone or equivalent; hydrocortisone, methylprednisolone, prednisolone, prednisone) |  |
| Category 5 | JAK inhibitors (tofacitinib, baricitinib,  cyclosporine) |  |
| **SLE medication categories^a^** | | |
| Category 1 | Hydroxychloroquine, chloroquine | Anti-malarial (non-immunosuppressing) |
| Category 2 | MTX | Less immunosuppressive |
|  | Azathioprine |  |
|  | Mycophenolate mofetil, mycophenolic acid |  |
|  | Tacrolimus, cyclosporine, voclosporin |  |
|  | Belimumab (subcutaneous) |  |
|  | Belimumab (intravenous) |  |
|  | Anifrolumab (intravenous) |  |
| Category 3 | Rituximab (intravenous) | Highly immunosuppressive |
|  | Cyclophosphamide (oral) |  |
|  | Cyclophosphamide (intravenous) |  |
| **MS medication categories^a^** | | |
| Category 1 | Alemtuzumab (intravenous) | Highly effective and immunosuppressive |
|  | Cladribine (oral) |  |
|  | Mitoxantrone (intravenous) |  |
| Category 2 | Rituximab (intravenous) | Highly effective and immunosuppressive, anti-CD20 |
|  | Ocrelizumab (intravenous) |  |
|  | Ofatumumab (subcutaneous) |  |
| Category 3 | Fingolimod, siponimod, ozanimod, ponesimod | Highly effective and immunosuppressing, SP1 receptor modulators |
| Category 4 | Natalizumab (intravenous) | Highly effective and less immunosuppressive therapies |
| Category 5 | Fumarates: dimethyl fumarate, diroximel fumarate, monomethyl fumarate | Less effective and less immunosuppressive therapies |
|  | Glatiramer acetate (subcutaneous or intramuscular) |  |
|  | Intravenous IgG |  |
|  | Interferon beta (subcutaneous or intramuscular): recombinant human interferon beta-1b, recombinant human interferon-1a, pegylated recombinant interferon beta-1a |  |
|  | Teriflunomide |  |
|  | Azathioprine, MTX, mycophenolate mofetil (all uncommonly used) |  |
| **PsO medication categories** | | |
| Category 1  (least severe) | Topical medications (topical corticosteroids, calcineurin inhibitors, vitamin D analogues, tazarotene, salicylic acid, anthralin, coal tar/liquor carbonis detergens, roflumilast, tapinarof), or no treatment from categories 2–5 | Medication categories for PsO were determined by the highest category of medication prescribed during the search periods specified below:  • Biologic DMARDs, intralesional/intramuscular corticosteroids, psoralen and ultraviolet A, photodynamic therapy, narrowband ultraviolet B (with or without tar), ultraviolet phototherapy (with or without tar), and excimer laser (3 months before index date)  • Targeted synthetic DMARDs (JAK inhibitors) (3 months before index date to 1 month after index date)  • NSAIDs, topical medications, steroids, and conventional synthetic DMARDs (medication prescribed/refilled within 6 months prior to index date)  Individuals who meet the criteria for both PsO and PsA were included in both the PsO and PsA cohorts. The most advanced medication category used for treatment of either condition was used for both cohorts. |
| Category 2 | Psoralen and ultraviolet A, photodynamic therapy, narrowband ultraviolet B (with or without tar), ultraviolet phototherapy (with or without tar), excimer laser. |  |
| Category 3 | Conventional synthetic DMARDs (traditional DMARDs [MTX, cyclosporine, acitretin, apremilast, azathioprine, hydroxyurea, mycophenolate mofetil, tacrolimus, leflunomide, thioguanine], intralesional/intramuscular corticosteroids [triamcinolone]). |  |
| Category 4 | Biologic DMARDs (administered either subcutaneous or intravenous, TNF-α inhibitors [etanercept, infliximab and biosimilars, adalimumab and biosimilars, certolizumab pegol, golimumab], IL-12/IL-23 inhibitors [ustekinumab], IL-17 inhibitors [secukinumab, ixekizumab, brodalumab, bimekizumab], IL-23 inhibitors [guselkumab, tildrakizumab-asmn, risankizumab-rzaa]). |  |
| Category 5  (most severe) | Targeted synthetic DMARDs (JAK inhibitors [tofacitinib, upadacitinib, deucravacitinib]). |  |
| **PsA medication categories** | | |
| Category 1  (least severe) | Non-pharmacologic therapies, or no treatment from categories 2–5. | Medication categories for PsA were determined by the highest category of medication prescribed during the search periods specified below:  • Biologic DMARDs, intralesional/intramuscular corticosteroids, psoralen and ultraviolet A, photodynamic therapy, narrowband ultraviolet B (with or without tar), ultraviolet phototherapy (with or without tar), and excimer laser (3 months before index date)  • Targeted synthetic DMARDs (JAK inhibitors) (3 months before index date to 1 month after index date)  • NSAIDs, topical medications, steroids, and conventional synthetic DMARDs (medication prescribed/refilled within 6 months prior to index date)  Individuals who meet the criteria for both PsO and PsA were included in both the PsO and PsA cohorts. The most advanced medication category used for treatment of either condition was used for both cohorts. |
| Category 2 | NSAIDs (ibuprofen, naproxen, indomethacin, meloxicam, celecoxib, nabumetone, etodolac, diclofenac, sulindac, salsalate, ketorolac), low-dose steroids (methylprednisolone, prednisolone, prednisone, <5 mg prednisone or equivalent). |  |
| Category 3 | Conventional synthetic DMARDs (oral small molecules [MTX, sulfasalazine, leflunomide, apremilast], high-dose systemic steroids [hydrocortisone, methylprednisolone, prednisolone, triamcinolone, prednisone, ≥5 mg prednisone or equivalent]). |  |
| Category 4 | Biologic DMARDs (TNF-α inhibitors [etanercept, infliximab and biosimilars, adalimumab and biosimilars, certolizumab pegol, golimumab], IL-12/IL-23 inhibitors [ustekinumab], IL-17 inhibitors [secukinumab, ixekizumab], CTLA4-immunoglobulin [abatacept], IL-23 inhibitors [guselkumab, risankizumab-rzaa and biosimilars]). |  |
| Category 5^b^ | Targeted synthetic DMARDs (JAK inhibitors [tofacitinib, upadacitinib and biosimilars]). |  |

^a^Infusion therapies dosed monthly are still considered an active treatment if they have been received in the past 90 days to account for interruptions in treatment.

^b^Categories 1–5 were used as listed, as the risk of HZ is greater in those treated with JAK inhibitors compared to the risk in those treated with medications in category 4.

5-ASA, 5-aminosalicylate; CD20, cluster of differentiation 20; CTLA4, cytotoxic T-lymphocyte associated protein 4; DMARDs, disease-modifying antirheumatic drugs; HZ, herpes zoster; IBD, inflammatory bowel disease; IgG, immunoglobulin G; IL, interleukin; JAK, Janus kinase; MS, multiple sclerosis; MTX, methotrexate; NSAIDs, non-steroidal anti-inflammatory drugs; PsA, psoriatic arthritis; PsO, psoriasis; RA, rheumatoid arthritis; SLE, systemic lupus erythematosus; SP1; sphingosine 1-phosphate, TNF- α, tumor necrosis factor alpha.

## Supplementary Table 2: Inclusion and exclusion criteria

| **Inclusion criteria** |
| --- |
| - Individuals aged ≥50 years at the index date for all study objectives and registered as beneficiary in the Optum® database. |
| - Individuals meeting the diagnosis criteria for RA, IBD, SLE, MS, PsO, or PsA prior to the index date.^a^ |
| - Individuals who have received the first dose of RZV on or after 1 January 2018. |
| - Individuals with 365 days of continuous enrollment (allowing administrative gaps 30 days) prior to the index date (baseline period) and continuous enrollment in the 30 days after the index date. |
| **Exclusion criteria** |
| - Any previous RZV doses before index date (for unvaccinated participants only) using all available data. |
| - Receipt of a second dose of RZV less than 28 days apart, since the Advisory Committee on Immunization Practices (ACIP) guidelines state that these participants must repeat the second dose [1] |
| - Receipt of a live zoster vaccine (ZVL) any time during the baseline, as this may affect rates of HZ. |
| - A HZ diagnosis or prescription fills for oral acyclovir, valacyclovir, or famciclovir given specifically for HZ and within 30 days of index date, since it is unclear if the HZ episode began before or after the index date and whether the length of time since vaccination (for RZV vaccinated participants) is long enough to allow for sufficient development of immunity. |
| - A HZ diagnosis or prescription fills for oral acyclovir, valacyclovir, or famciclovir in the 12 months before the index date to ensure that HZ diagnoses after the index date are new, rather than carried over from HZ episodes prior to the index date. |
| - Postherpetic neuralgia diagnosis in the 12 months before the index date. |
| - Censoring events within 30 days after the index date (before the start of follow-up):   - HZ occurrence.   - End of continuous enrolment (period of uninterrupted insurance coverage with gap allowance of 30 days).   - Death (date of death including the year and month of death).   - End of data availability/study period (i.e., 31 December 2021).   - Receipt of RZV (additional dose for vaccinated participants or first RZV dose in the case of unvaccinated participants):   -For two-dose cohort, vaccinated participants were censored upon receipt of a third dose.  -For one-dose cohort, vaccinated participants were censored upon receipt of a second dose.   - - Receipt of ZVL vaccination. |

^a^Diagnosis criteria for the AIDs are detailed in **Supplementary table 3**.

AID, autoimmune disease; HZ, herpes zoster; IBD, inflammatory bowel disease; MS, multiple sclerosis; PsA, psoriatic arthritis; PsO, psoriasis; RA, rheumatoid arthritis; RZV, adjuvanted recombinant zoster vaccine; SLE, systemic lupus erythematosus.

**Supplementary references**

1. Dooling KL, Guo A, Patel M, et al. Recommendations of the Advisory Committee on Immunization Practices for Use of Herpes Zoster Vaccines. MMWR Morb Mortal Wkly Rep **2018**; 67:103-8.

## Supplementary table 3: Diagnosis criteria for selected AIDs

| **RA** |
| --- |
| At least one inpatient claim with a diagnosis code for RA (ICD-10: M05, M06)  OR  ≥2 physician outpatient claims with RA diagnosis during an office visit and/or emergency visit that were at least 30 days apart in the 365-day baseline period prior to the index date.  The algorithm that includes ≥2 physician outpatient claims with RA diagnosis during an office visit and/or emergency visit has been used extensively in the literature though no PPV has been reported [1]. |
| **IBD (including CD and UC)** |
| At least one inpatient claim with a diagnosis code for CD (ICD-10: K50) and UC (ICD-10: K51)  OR  ≥2 physician outpatient claims with CD (ICD-10: K50) and UC (ICD-10: K51) diagnosis during an office visit and/or emergency visit that were at least 30 days apart in the 365-day baseline period prior to the index date.  The algorithm that includes ≥2 physician outpatient claims with CD and UC diagnoses during an office visit and/or emergency visit has been used extensively in the literature though no PPV has been reported [2].  Algorithms that have been published to classify mutually exclusive groups of UC and CD patients will be considered to differentiate between the two conditions [3]. Patients were identified as having UC if (i) they had more UC-related patient admissions than CD-related inpatient admissions; (ii) they had an equal number of UC- and CD-related inpatient admissions but more UC-related outpatient visits than CD-related outpatient visits; (iii) they had an equal number of UC- and CD-related inpatient admissions and outpatient visits [4,5]. |
| **SLE** |
| At least one inpatient claim with a diagnosis code for SLE (ICD-10: M32.1, M32.8, M32.9)  OR  ≥2 physician outpatient claims with SLE diagnosis during an office visit and/or emergency visit that were at least 30 days apart in the 365-day baseline period prior to the index date  OR  >1 rheumatologist visit/encounter/claim for SLE.  The algorithm that includes ≥1 rheumatologist visit/encounter/claim has demonstrated a PPV of 95% and sensitivity of 83% [6]. |
| **MS** |
| Three or more of any combination of inpatient diagnoses (any position) of MS (ICD-10: G35), ambulatory visit diagnoses of MS, emergency department diagnoses of MS, or MS-specific disease-modifying therapy fills/infusions during the 365-day baseline period. At least one of these must be an inpatient, ambulatory visit, or emergency department diagnosis of MS.  This algorithm has demonstrated a PPV of 95%–97% and sensitivity of 85%–93% [7]. |
| **PsO** |
| At least one inpatient claim with a diagnosis code for PsO (ICD-10: L40)  OR  ≥2 physician outpatient claims with PsO diagnosis during an office visit and/or emergency visit that were at least 30 days apart  OR  ≥1 dermatologist visit/encounter/claim for PsO in the 365-day baseline period prior to the index date.  The algorithm that includes ≥1 dermatologist visit/encounter/claim has demonstrated a PPV of 90% and sensitivity of 88% [8]. These algorithms assume PsO without PsA. Based on the rapid data queries, 9% of patients diagnosed with PsO had PsA. In practice, rheumatologists tend to assess PsO and PsA by criteria which are unique to each. Then, they assess the two AIDs together in patients with symptoms of both. Sensitivity analyses were performed on patients who had either PsO or PsA, patients who had PsO only, patients who had PsA only, and patients who had both PsO and PsA to address the potential overlap between PsO and PsA. |
| **PsA** |
| At least one inpatient claim with a diagnosis code for PsA (ICD-10: L40.5)  OR  ≥2 physician outpatient claims with PsA diagnosis during an office visit and/or emergency visit that were at least 30 days apart  OR  ≥2 rheumatologist visit/encounter/claim for PsA  OR  ≥1 rheumatologist diagnosis code for PsA together with ≥1 dermatologist diagnosis code for PsA in the 365-day baseline period prior to the index date.  The algorithm that includes ≥2 rheumatologist visit/encounter/claim for PsA has demonstrated a PPV of 81% and sensitivity of 77% [8]. These algorithms assume PsA without PsO. Based on the rapid data queries, 37% of patients diagnosed with PsA had PsO. Sensitivity analyses were conducted to address the potential overlap between PsO and PsA. |

**Note:** Claims based algorithms were obtained from the published literature defining AID conditions and were validated with demonstrated PPV. Such algorithms were used to identify AIDs. If patients met >1 AID definitions, they were included in multiple cohorts; AIDs were analyzed separately.

AID, autoimmune disease; CD, Crohn’s disease; ICD-10, International Classification of Diseases, 10^th^ Revision; IBD, inflammatory bowel disease; MS, multiple sclerosis; PPV, positive predictive value; PsA, psoriatic arthritis; PsO, psoriasis; RA, rheumatoid arthritis; SLE, systemic lupus erythematosus; UC, ulcerative colitis.

**Supplementary references**

1 MacLean CH, Louie R, Leake B, et al. Quality of care for patients with rheumatoid arthritis. JAMA **2000**; 284:984-92.

2 Weng X, Liu L, Barcellos LF, Allison JE, Herrinton LJ. Clustering of inflammatory bowel disease with immune mediated diseases among members of a Northern California-managed care organization. Am J Gastroenterol **2007**; 102:1429-35.

3 Pilon D, Ding Z, Muser E, et al. Long-term direct and indirect costs of ulcerative colitis in a privately-insured United States population. Curr Med Res Opin **2020***;* 36:1285-94.

4 Bernstein CN, Blanchard JF, Rawsthorne P, Wajda A. Epidemiology of Crohn's disease and ulcerative colitis in a central Canadian province: a population-based study. Am J Epidemiol **1999**; 149:916-24.

5 Shaw SY, Blanchard JF, Bernstein CN. Association between the use of antibiotics and new diagnoses of Crohn's disease and ulcerative colitis. Am J Gastroenterol **2011**; 106: 2133-42.

6 Hanly JG. Diagnosis and management of neuropsychiatric SLE. Nat Rev Rheumatol **2014**; 10:338-47.

7 Wallin MT, Culpepper WJ, Campbell JD, et al. The prevalence of MS in the United States: A population-based estimate using health claims data. Neurology 2019; 92:e1029-40.

8 Asgari MM, Wu JJ, Gelfand JM, et al. Validity of diagnostic codes and prevalence of psoriasis and psoriatic arthritis in a managed care population, 1996-2009. Pharmacoepidemiol Drug Saf **2013**; 22:842-9.

## Supplementary Table 4: Baseline characteristics of the patient population in the one-dose cohort after matching by vaccination status

| **Characteristic** | **RZV cohort**  **N=44,865** | **Non-RZV cohort**  **N=130,458** |
| --- | --- | --- |
| **Mean age at index date (min–max), years** | 68.7 (50–90) | 68.9 (50–90) |
| **Age group; n (%)** |  |  |
| 50–59 years | 7,590 (16.9) | 21,764 (16.7) |
| 60–69 years | 15,733 (35.1) | 45,444 (34.8) |
| 70–79 years | 16,330 (36.4) | 47,875 (36.7) |
| ≥80 years | 5,212 (11.6) | 15,375 (11.8) |
| **AID condition; n (%)** |  |  |
| RA | 19,016 (42.4) | 55,034 (42.2) |
| IBD | 7,461 (16.6) | 22,396 (17.2) |
| SLE | 2,294 (5.1) | 6,626 (5.1) |
| MS | 2,752 (6.1) | 7,899 (6.1) |
| PsO | 10,815 (24.1) | 31,144 (23.9) |
| PsA | 2,527 (5.6) | 7,359 (5.6) |
| **Sex; n (%)** |  |  |
| Female | 29,492 (65.7) | 85,305 (65.4) |
| **Race/Ethnicity; n (%)** |  |  |
| Asian | 1,275 (2.8) | 3,490 (2.7) |
| Black | 4,102 (9.1) | 11,749 (9.0) |
| Hispanic | 4,093 (9.1) | 11,799 (9.0) |
| White | 35,395 (78.9) | 103,420 (79.3) |
| **Region of residence within the United States; n (%)** |  |  |
| Midwest | 10,919 (24.3) | 32,103 (24.6) |
| Northeast | 5,356 (11.9) | 15,047 (11.5) |
| South | 18,445 (41.1) | 53,116 (40.7) |
| West | 10,145 (22.6) | 30,192 (23.1) |
| **At least one comorbidity^a^; n (%)** | 26,643 (59.4) | 77,418 (59.3) |
| **Years of follow-up; mean** | 0.2 | 0.4 |
| **Years of follow-up; median (IQR)** | 0.2 (0.1; 0.4) | 0.5 (0.5; 0.5) |
| **At least one concomitant vaccination; n (%)** | 5,029 (11.2) | 9,915 (7.6) |
| **Medication category^b, c^; n (%)** |  |  |
| Category 1 | 19,012 (42.4) | 54,994 (42.2) |
| Category 2 | 8,222 (18.3) | 23,978 (18.4) |
| Category 3 | 5,125 (11.4) | 14,992 (11.5) |
| Category 4 | 11,729 (26.1) | 34,225 (26.2) |
| Category 5 | 777 (1.7) | 2,269 (1.7) |

^a^Comorbidities were identified through International Classification of Diseases, 10th Revision (ICD-10) diagnosis codes. Following comorbidities were considered:

Chronic diseases: Kidney disease (ICD-10 codes: I12.0, I12.9, I13.1*, N03.2-N03.7, N05.2-N05.7, N18.*, N19.*, N25.0, Z49.0*-Z49.2*, Z94.0, Z99.2); cardiovascular disease (ICD-10 codes: I21.*, I22.*, I25.2, I09.9, I11.0, I13.0, I13.2, I25.5, I42.0, I42.5-I42.9, I43.*, I50.*, P29.0); pulmonary disease (ICD-10 codes: I27.8*, I27.9, J40.*-J47.*, J60.*-J67.*, J68.4, J70.1, J70.3); liver disease (ICD-10 codes: B18.*, I85.0*, I86.4, I98.2, K70.0, K70.1*-K70.4*, K70.9, K71.1*, K71.3, K71.4, K71.5*, K71.7, K72.1*, K72.9*, K73.*, K74.*, K76.0, K76.2-K76.7, K76.8*, K76.9, Z94.4); diabetes mellitus (ICD-10 codes: E10.*-E14.*).

AIDs: RA (ICD-10 codes: M05.*, M06.*); IBD (ICD-10 codes: K50.*, K51.*); SLE (ICD-10 codes: M32.1, M32.8, M32.9); MS (ICD-10 code: G35); PsO/PsA (ICD-10 codes: L40.*, L40.5*).

Immunosuppressant conditions: Lymphoma/leukemia (ICD-10 codes: C81.*C86.*, C88.*, C90.*-C96.*, D45, D46.*); congenital and other immunodeficiencies (ICD-10 codes: D61.09, D61.3, D61.82, D61.9, D70.0, D71, D80.0, D80.1, D80.5, D80.8, D81.*, D82.*, D83.*, D84.0, D84.1, D84.89, D84.9, D89.81*, D89.82, D89.9, E31.0, E70.330, G11.3, Q82.4, Q89.0*); asplenia/hyposplenia (ICD-10 codes: D57.00, D57.01, D57.02, D57.1, D57.2, D57.20, D57.21, D57.211, D57.212, D57.219, D57.4, D57.40, D57.41, D57.411, D57.412, D57.419, D57.8, D57.80, D57.81, D57.811, D57.812, D57.819, D73.0, Q89.01, Q89.09, Z90.81).

SARS-CoV-2 infection (ICD-10 code: U07.1) and pneumonia due to COVID-19 (ICD-10 code: J12.82).

^b^Medication categories are detailed in **Supplementary Table 1**.

^c^The cutoff for steroid use is 5 mg for ≥93% of the patients with RA (7,546 [93.8%] vaccinated patients; 21,940 [93.5%] unvaccinated patients), IBD (1,664 [98.2%] vaccinated patients; 5,245 [98.1%] unvaccinated patients), or PsA (604 [95.3%] vaccinated patients; 1,824 [94.8%] unvaccinated patients).

AID, autoimmune disease; IBD, inflammatory bowel disease; IQR, interquartile range; MS, multiple sclerosis; N, total number of patients; n (%), number/percentage of patients in each category; non-RZV cohort, unvaccinated patients; PsA, psoriatic arthritis; PsO, psoriasis; RA, rheumatoid arthritis; RZV, adjuvanted recombinant zoster vaccine; RZV cohort, RZV-vaccinated patients; SARS-CoV-2, severe acute respiratory syndrome coronavirus 2; SLE, systemic lupus erythematosus.

## Supplementary Table 5: VE against HZ after two RZV doses in patients with selected AIDs, stratified by age, sex, time interval between two RZV doses, medication category based on current use at the index date (mutually exclusive), and time since vaccination

| **AID** | | **Characteristic** | | **N** | **VE (95% CIs)** |
| --- | --- | --- | --- | --- | --- |
| **RA** | | Age group (years) | 50–59 | 6,699 | 76.0 (52.5; 87.9) |
|  |  |  | 60–69 | 16,945 | 60.5 (45.6; 71.3) |
|  |  |  | 70–79 | 19,250 | 60.8 (48.7; 70.1) |
|  |  |  | ≥80 | 7,499 | 63.8 (40.7; 77.9) |
|  |  |  |  |  |  |
|  |  | Sex | Female | 36,380 | 62.1 (53.4; 69.2) |
|  |  |  | Male | 13,264 | 64.9 (48.0; 76.4) |
|  |  |  |  |  |  |
|  |  | Time interval between two RZV doses (days) | 28–59 | 1,332 | 68.9 (−34.1; 92.8) |
|  |  |  | 60–179 | 43,707 | 61.1 (52.9; 67.9) |
|  |  |  | 180–<365 | 6,631 | 75.7 (52.0; 87.7) |
|  |  |  |  |  |  |
|  |  | Medication use^a^ | Category 1 | 11,737 | 72.3 (52.0; 84.0) |
|  |  |  | Category 2 | 14,676 | 58.5 (41.6; 70.6) |
|  |  |  | Category 3 | 4,572 | 53.7 (18.2; 73.7) |
|  |  |  | Category 4 | 19,413 | 63.9 (52.0; 72.8) |
|  |  |  | Category 5 | 1,781 | 65.6 (33.4; 82.2) |
|  |  |  |  |  |  |
|  |  | Time since vaccination (years) | 0−<1 | 49,644 | 68.4 (58.8; 75.8) |
|  |  |  | 1−<2 | 32,304 | 56.8 (41.3; 68.2) |
|  |  |  | 2−<3 | 15,658 | 59.5 (33.1; 75.4) |
|  |  |  | ≥3 | 3,760 | −10.8 (−268.1; 66.6)^b^ |
| **IBD** | | Age group (years) | 50–59 | 3,669 | 74.8 (29.9; 90.9) |
|  |  |  | 60–69 | 7,078 | 76.4 (45.7; 89.8) |
|  |  |  | 70–79 | 8,012 | 73.0 (53.2; 84.4) |
|  |  |  | ≥80 | 2,683 | 68.0 (9.7; 88.6) |
|  |  |  |  |  |  |
|  |  | Sex | Female | 11,571 | 80.2 (65.2; 88.7) |
|  |  |  | Male | 9,514 | 62.3 (35.2; 78.0) |
|  |  |  |  |  |  |
|  |  | Time interval between two RZV doses (days) | 28–59 | 797 | 100 (.; 100) |
|  |  |  | 60–179 | 19,422 | 75.1 (61.9; 83.7) |
|  |  |  | 180–<365 | 3,535 | 52.9 (−18.9; 81.3) |
|  |  |  |  |  |  |
|  |  | Medication use^a^ | Category 1 | 7,187 | 63.1 (26.0; 81.6) |
|  |  |  | Category 2 | 5,810 | 80.7 (52.0; 92.2) |
|  |  |  | Category 3 | 3,364 | 57.2 (9.9; 79.7) |
|  |  |  | Category 4 | 5,844 | 86.0 (65.7; 94.3) |
|  |  |  | Category 5 | 59 | 41.9 (−460.4; 94.0) |
|  |  |  |  |  |  |
|  |  | Time since vaccination (years) | 0−<1 | 21,085 | 66.7 (45.1; 79.9) |
|  |  |  | 1−<2 | 13,819 | 77.3 (53.3; 89.0) |
|  |  |  | 2−<3 | 6,700 | 79.3 (32.7; 93.6) |
|  |  |  | ≥3 | 1,652 | 100 (.; 100) |
|  | **UC** | Age group (years) | 50–59 | 1,945 | 51.8 (−65.6; 86.0) |
|  |  |  | 60–69 | 4,096 | 64.4 (9.4; 86.0) |
|  |  |  | 70–79 | 4,982 | 66.5 (37.2; 82.1) |
|  |  |  | ≥80 | 1,729 | 59.0 (−38.6; 87.9) |
|  |  |  |  |  |  |
|  |  | Sex | Female | 6,655 | 78.2 (55.0; 89.4) |
|  |  |  | Male | 5,892 | 41.1 (−5.1; 67.0) |
|  |  |  |  |  |  |
|  |  | Time interval between two RZV doses (days) | 28–59 | 318 | 100 (.; 100) |
|  |  |  | 60–179 | 11,235 | 66.7 (45.6; 79.6) |
|  |  |  | 180–<365 | 1,526 | 22.0 (−137.1; 74.3) |
|  |  |  |  |  |  |
|  |  | Medication use^a^ | Category 1 | 4,358 | 51.6 (−8.2; 78.4) |
|  |  |  | Category 2 | 4,179 | 74.9 (36.9; 90.0) |
|  |  |  | Category 3 | 1,377 | 13.4 (−116.8; 65.4) |
|  |  |  | Category 4 | 3,199 | 83.8 (47.9; 94.9) |
|  |  |  | Category 5 | 56 | 13.9 (−852.9; 92.2) |
|  |  |  |  |  |  |
|  |  | Time since vaccination (years) | 0−<1 | 12,547 | 58.8 (24.4; 77.5) |
|  |  |  | 1−<2 | 8,372 | 65.3 (23.6; 84.3) |
|  |  |  | 2−<3 | 4,125 | 63.8 (−22.2; 89.3) |
|  |  |  | ≥3 | 997 | 100 (.; 100) |
|  | **CD** | Age group (years) | 50–59 | 1,810 | 90.4 (29.5; 98.7) |
|  |  |  | 60–69 | 3,153 | 92.0 (41.3; 98.9) |
|  |  |  | 70–79 | 3,209 | 80.6 (46.3; 93.0) |
|  |  |  | ≥80 | 1,002 | 81.8 (−37.9; 97.6) |
|  |  |  |  |  |  |
|  |  | Sex | Female | 5,159 | 83.9 (60.4; 93.5) |
|  |  |  | Male | 3,874 | 88.7 (53.4; 97.3) |
|  |  |  |  |  |  |
|  |  | Time interval between two RZV doses (days) | 28–59 | 263 | 100 (.; 100) |
|  |  |  | 60–179 | 8,003 | 88.2 (71.0; 95.2) |
|  |  |  | 180–<365 | 1,199 | 61.8 (−68.1; 91.3) |
|  |  |  |  |  |  |
|  |  | Medication use^a^ | Category 1 | 2,906 | 80.9 (19.8; 95.4) |
|  |  |  | Category 2 | 1,738 | 85.2 (−11.0; 98.0) |
|  |  |  | Category 3 | 2,107 | 84.5 (35.2; 96.3) |
|  |  |  | Category 4 | 2,775 | 89.6 (57.4; 97.5) |
|  |  |  | Category 5 | 6 | 100 (.; 100) |
|  |  |  |  |  |  |
|  |  | Time since vaccination (years) | 0−<1 | 9,033 | 79.1 (48.1; 91.6) |
|  |  |  | 1−<2 | 5,762 | 87.8 (49.8; 97.1) |
|  |  |  | 2−<3 | 2,711 | 100 (.; 100) |
|  |  |  | ≥3 | 676 | 100 (.; 100) |
| **SLE** | | Age group (years) | 50–59 | 1,304 | −133.9 (−667.1; 28.7)^b^ |
|  |  |  | 60–69 | 2,298 | 70.9 (18.6; 89.6) |
|  |  |  | 70–79 | 2,035 | 65.8 (3.0; 87.9) |
|  |  |  | ≥80 | 505 | 87.0 (3.4; 98.3) |
|  |  |  |  |  |  |
|  |  | Sex | Female | 5,386 | 53.1 (17.4; 73.4) |
|  |  |  | Male | 678 | 100 (.; 100) |
|  |  |  |  |  |  |
|  |  | Time interval between two RZV doses (days) | 28–59 | 163 | 100 (.; 100) |
|  |  |  | 60–179 | 5,250 | 55.8 (22.2; 74.9) |
|  |  |  | 180–<365 | 878 | 100 (.; 100) |
|  |  |  |  |  |  |
|  |  | Medication use^a^ | Category 1 | 4,749 | 63.2 (28.7; 81) |
|  |  |  | Category 2 | 1,339 | 48.1 (−51.2; 82.2) |
|  |  |  | Category 3 | 39 | 100 (.; 100) |
|  |  |  | Category 4 | NA | NA (NA; NA) |
|  |  |  | Category 5 | NA | NA (NA; NA) |
|  |  |  |  |  |  |
|  |  | Time since vaccination (years) | 0−<1 | 6,064 | 55.0 (9.0; 77.7) |
|  |  |  | 1−<2 | 3,730 | 81.9 (24.1; 95.7) |
|  |  |  | 2−<3 | 1,681 | 23.7 (−181.8; 79.3) |
|  |  |  | ≥3 | 396 | 100 (.; 100) |
| **MS** | | Age group (years) | 50–59 | 2,199 | 46.2 (−56.8; 81.5) |
|  |  |  | 60–69 | 3,385 | 18.7 (−65.1; 59.9) |
|  |  |  | 70–79 | 1,905 | 65.4 (−15.7; 89.6) |
|  |  |  | ≥80 | 296 | 100 (.; 100) |
|  |  |  |  |  |  |
|  |  | Sex | Female | 5,784 | 50.9 (11.2; 72.8) |
|  |  |  | Male | 1,891 | 35.1 (−94.4; 78.3) |
|  |  |  |  |  |  |
|  |  | Time interval between two RZV doses (days) | 28–59 | 230 | −101.5 (−2,206.9; 82.4)^b^ |
|  |  |  | 60–179 | 6,722 | 43.2 (0.8; 67.5) |
|  |  |  | 180–<365 | 1,031 | 81.6 (−39.0; 97.6) |
|  |  |  |  |  |  |
|  |  | Medication use^a^ | Category 1 | 6,744 | 44.7 (5.1; 67.7) |
|  |  |  | Category 2 | 163 | 100 (.; 100) |
|  |  |  | Category 3 | 256 | 7.2 (−792.2; 90.4) |
|  |  |  | Category 4 | 565 | 100 (.; 100) |
|  |  |  | Category 5 | 18 | 0.0 (0.0; 0.0) |
|  |  |  |  |  |  |
|  |  | Time since vaccination (years) | 0−<1 | 7,675 | 48.6 (−9.3; 75.8) |
|  |  |  | 1−<2 | 4,670 | 62.7 (−6.3; 86.9) |
|  |  |  | 2−<3 | 2,224 | 9.7 (−153.3; 67.8) |
|  |  |  | ≥3 | 471 | 100 (.; 100) |
| **PsO** | | Age group (years) | 50–59 | 4,980 | 49.3 (−8.0; 76.2) |
|  |  |  | 60–69 | 10,622 | 82.6 (62.6; 91.9) |
|  |  |  | 70–79 | 11,191 | 77.4 (59.3; 87.5) |
|  |  |  | ≥80 | 3,242 | 92.6 (46.0; 99.0) |
|  |  |  |  |  |  |
|  |  | Sex | Female | 15,843 | 75.7 (60.6; 85.0) |
|  |  |  | Male | 13,827 | 79.5 (60.9; 89.2) |
|  |  |  |  |  |  |
|  |  | Time interval between two RZV doses (days) | 28–59 | 812 | 100 (.; 100) |
|  |  |  | 60–179 | 26,547 | 79.1 (68.1; 86.4) |
|  |  |  | 180–<365 | 3,340 | 42.1 (−51.8; 77.9) |
|  |  |  |  |  |  |
|  |  | Medication use^a^ | Category 1 | 20,836 | 76.1 (61.4; 85.3) |
|  |  |  | Category 2 | 470 | 100 (.; 100) |
|  |  |  | Category 3 | 4,937 | 92.0 (67.4; 98.0) |
|  |  |  | Category 4 | 4,040 | 62.2 (16.5; 82.9) |
|  |  |  | Category 5 | 81 | −4.8 (−1057.5; 90.5)^b^ |
|  |  |  |  |  |  |
|  |  | Time since vaccination (years) | 0−<1 | 29,670 | 78.9 (64.4; 87.5) |
|  |  |  | 1−<2 | 18,576 | 81.2 (59.6; 91.3) |
|  |  |  | 2−<3 | 8,703 | 64.6 (−1.3; 87.7) |
|  |  |  | ≥3 | 2,089 | 56.1 (−275.4; 94.9) |
| **PsA** | | Age group (years) | 50–59 | 1,552 | −9.2 (−323.3; 71.8)^b^ |
|  |  |  | 60–69 | 2,897 | 81.0 (38.6; 94.1) |
|  |  |  | 70–79 | 2,272 | 52.2 (−14.0; 80.0) |
|  |  |  | ≥80 | 447 | 100 (.; 100) |
|  |  |  |  |  |  |
|  |  | Sex | Female | 3,814 | 60.7 (23.6; 79.8) |
|  |  |  | Male | 3,237 | 79.0 (11.4; 95.0) |
|  |  |  |  |  |  |
|  |  | Time interval between two RZV doses (days) | 28–59 | 217 | 100 (.; 100) |
|  |  |  | 60–179 | 6,220 | 62.1 (28.7; 79.9) |
|  |  |  | 180–<365 | 939 | 80.8 (−46.0; 97.5) |
|  |  |  |  |  |  |
|  |  | Medication use^a^ | Category 1 | 1,998 | 42.8 (−67.4; 80.4) |
|  |  |  | Category 2 | 809 | 80.1 (−51.3; 97.4) |
|  |  |  | Category 3 | 1,870 | 91.4 (36.8; 98.8) |
|  |  |  | Category 4 | 2,445 | 48.6 (−34.5; 80.3) |
|  |  |  | Category 5 | 205 | 27.8 (−594.7; 92.5) |
|  |  |  |  |  |  |
|  |  | Time since vaccination (years) | 0−<1 | 7,051 | 57.6 (10.8; 79.8) |
|  |  |  | 1−<2 | 4,348 | 75.6 (20.2; 92.5) |
|  |  |  | 2−<3 | 1,946 | 41.7 (−421.5; 93.5) |
|  |  |  | ≥3 | 444 | 100 (.; 100) |
| **PsO or PsA** | | Age group (years) | 50–59 | 6,108 | 40.5 (−14.5; 69.1) |
|  |  |  | 60–69 | 12,667 | 82.3 (66.4; 90.7) |
|  |  |  | 70–79 | 12,810 | 72.7 (55.7; 83.2) |
|  |  |  | ≥80 | 3,585 | 94.3 (58.5; 99.2) |
|  |  |  |  |  |  |
|  |  | Sex | Female | 18,566 | 71.9 (58.5; 80.9) |
|  |  |  | Male | 16,079 | 79.7 (63.4; 88.7) |
|  |  |  |  |  |  |
|  |  | Time interval between two RZV doses (days) | 28–59 | 997 | 100 (.; 100) |
|  |  |  | 60–179 | 31,027 | 75.7 (65.5; 82.9) |
|  |  |  | 180–<365 | 4,149 | 57.6 (−0.3; 82.0) |
|  |  |  |  |  |  |
|  |  | Medication use^a^ | Category 1 | NA | NA (NA; NA) |
|  |  |  | Category 2 | NA | NA (NA; NA) |
|  |  |  | Category 3 | NA | NA (NA; NA) |
|  |  |  | Category 4 | NA | NA (NA; NA) |
|  |  |  | Category 5 | NA | NA (NA; NA) |
|  |  |  |  |  |  |
|  |  | Time since vaccination (years) | 0−<1 | 34,645 | 74.2 (60.5; 83.2) |
|  |  |  | 1−<2 | 21,764 | 79.8 (61.6; 89.4) |
|  |  |  | 2−<3 | 10,173 | 62.8 (4.3; 85.5) |
|  |  |  | ≥3 | 2,462 | 77.9 (−72.3; 97.2) |
| **PsO and PsA** | | Age group (years) | 50–59 | 856 | −25.9 (−592.3; 77.1)^b^ |
|  |  |  | 60–69 | 1,554 | 88.5 (15.1; 98.5) |
|  | |  | 70–79 | 1,170 | 67.4 (−41.8; 92.5) |
|  |  |  | ≥80 | 211 | 100 (.; 100) |
|  | |  |  |  |  |
|  |  | Sex | Female | 2,048 | 60.4 (−1.2; 84.5) |
|  | |  | Male | 1,712 | 100 (.; 100) |
|  |  |  |  |  |  |
|  | | Time interval between two RZV doses (days) | 28–59 | 116 | 100 (.; 100) |
|  |  |  | 60–179 | 3,315 | 73.6 (26.1; 90.5) |
|  | |  | 180–<365 | 421 | 60.4 (−222.5; 95.1) |
|  |  |  |  |  |  |
|  | | Medication use^a^ | Category 1 | NA | NA (NA; NA) |
|  | |  | Category 2 | NA | NA (NA; NA) |
|  | |  | Category 3 | NA | NA (NA; NA) |
|  | |  | Category 4 | NA | NA (NA; NA) |
|  | |  | Category 5 | NA | NA (NA; NA) |
|  | |  |  |  |  |
|  | | Time since vaccination (years) | 0−<1 | 3,760 | 70.7 (4.0; 91.1) |
|  |  |  | 1−<2 | 2,224 | 84.3 (−18.3; 97.9) |
|  | |  | 2−<3 | 942 | −14.9 (−1167.2; 89.6)^b^ |
|  |  |  | ≥3 | 186 | 100 (.; 100) |

^a^Medication categories are detailed in **Supplementary Table 1**.

^b^The VE is not reliable due to a small number of patients.

AID, autoimmune disease; CD, Crohn’s disease; CI, confidence interval; HZ, herpes zoster; IBD, inflammatory bowel disease; MS, multiple sclerosis; N, total number of patients; NA, not applicable; PsA, psoriatic arthritis; PsO, psoriasis; RA, rheumatoid arthritis; SLE, systemic lupus erythematosus; UC, ulcerative colitis; VE, vaccine effectiveness.

## Supplementary Table 6: VE against HZ after two RZV doses in patients with selected AIDs, stratified by age, sex, time interval between two RZV doses, medication category based on current use at the index date (mutually exclusive), and time since vaccination, with pooled categories (post-hoc analyses)

| **AID** | | **Characteristic** | | **N** | **VE (95% CIs)** |
| --- | --- | --- | --- | --- | --- |
| **All AIDs** | | Age group | 50–59 | 19,521 | 59.5 (41.6; 71.9) |
|  | |  | 60–69 | 41,203 | 66.2 (56.9; 73.5) |
|  |  |  | ≥70 | 56,835 | 67.9 (61.3; 73.4) |
|  |  |  |  |  |  |
|  |  | Sex | Female | 75,454 | 65.4 (59.4; 70.5) |
|  |  |  | Male | 40,613 | 68.9 (59.2; 76.2) |
|  |  |  |  |  |  |
|  |  | Time interval between two RZV doses (days) | 28–179 | 105,300 | 66.0 (60.7; 70.5) |
|  |  |  | 180–<365 | 15,164 | 69.7 (52.5; 80.7) |
|  |  |  |  |  |  |
|  |  | Medication use^a^ | Category 1 | 52,534 | 66.3 (56.9; 73.6) |
|  |  |  | Category 2 | 26,888 | 68.3 (57.5; 76.4) |
|  |  |  | Category 3–4 | 37,967 | 66.8 (58.4; 73.4) |
|  |  |  | Category 5 | 5,244 | 60.1 (36.7; 74.8) |
|  |  |  |  |  |  |
|  |  | Time since vaccination (years) | 0–<1 | 116,067 | 67.9 (61.2; 73.5) |
|  |  |  | 1–<2 | 74,450 | 67.3 (58.1; 74.5) |
|  |  |  | ≥2 | 35,677 | 57.7 (40.8; 69.8) |
| **RA** | | Age group | 50–59 | 6,699 | 76.0 (52.5; 87.9) |
|  |  |  | 60–69 | 16,945 | 60.5 (45.6; 71.3) |
|  |  |  | ≥70 | 26,561 | 61.6 (51.3; 69.7) |
|  |  |  |  |  |  |
|  |  | Sex | Female | 36,380 | 62.1 (53.4; 69.2) |
|  |  |  | Male | 13,264 | 64.9 (48.0; 76.4) |
|  |  |  |  |  |  |
|  |  | Time interval between two RZV doses (days) | 28–179 | 44,688 | 61.3 (53.1; 68.0) |
|  |  |  | 180–<365 | 6,631 | 75.7 (52.0; 87.7) |
|  |  |  |  |  |  |
|  |  | Medication use^a^ | Category 1 | 11,737 | 72.3 (52.0; 84.0) |
|  |  |  | Category 2 | 14,676 | 58.5 (41.6; 70.6) |
|  |  |  | Category 3–4 | 23,574 | 62.2 (51.3; 70.7) |
|  |  |  | Category 5 | 1,781 | 65.6 (33.4; 82.2) |
|  |  |  |  |  |  |
|  |  | Time since vaccination (years) | 0–<1 | 49,644 | 68.4 (58.8; 75.8) |
|  |  |  | 1–<2 | 32,304 | 56.8 (41.3; 68.2) |
|  |  |  | ≥2 | 15,658 | 54.2 (27.7; 71.0) |
| **IBD** | | Age group | 50–59 | 3,669 | 74.8 (29.9; 90.9) |
|  |  |  | 60–69 | 7,078 | 76.4 (45.7; 89.8) |
|  |  |  | ≥70 | 10,613 | 72.0 (54.5; 82.7) |
|  |  |  |  |  |  |
|  |  | Sex | Female | 11,571 | 80.2 (65.2; 88.7) |
|  |  |  | Male | 9,514 | 62.3 (35.2; 78.0) |
|  |  |  |  |  |  |
|  |  | Time interval between two RZV doses (days) | 28–179 | 19,231 | 76.0 (63.2; 84.3) |
|  |  |  | 180–<365 | 2,687 | 49.4 (−31.1; 80.4) |
|  |  |  |  |  |  |
|  |  | Medication use^a^ | Category 1 | 7,291 | 59.1 (20.7; 78.9) |
|  |  |  | Category 2 | 5,794 | 81.5 (54.1; 92.5) |
|  |  |  | Category 3–4 | 8,835 | 77.3 (59.2; 87.4) |
|  |  |  | Category 5 | 62 | 40.0 (−479.3; 93.8) |
|  |  |  |  |  |  |
|  |  | Time since vaccination (years) | 0–<1 | 21,085 | 66.7 (45.1; 79.9) |
|  |  |  | 1–<2 | 13,819 | 80.1 (57.2; 90.8) |
|  |  |  | ≥2 | 6,700 | 83.4 (46.6; 94.9) |
|  | **UC** | Age group | 50–59 | 1,945 | 51.8 (−65.6; 86.0) |
|  |  |  | 60–69 | 4,096 | 64.4 (9.4; 86.0) |
|  |  |  | ≥70 | 6,665 | 65.1 (39.0; 80.0) |
|  |  |  |  |  |  |
|  |  | Sex | Female | 6,655 | 78.2 (55.0; 89.4) |
|  |  |  | Male | 5,892 | 41.1 (−5.1; 67.0) |
|  |  |  |  |  |  |
|  |  | Time interval between two RZV doses (days) | 28–179 | 11,470 | 67.4 (46.8; 80.0) |
|  |  |  | 180–<365 | 1,526 | 22.0 (−137.1; 74.3) |
|  |  |  |  |  |  |
|  |  | Medication use^a^ | Category 1 | 4,408 | 52.6 (−5.9; 78.8) |
|  |  |  | Category 2 | 4,195 | 76.1 (40.2; 90.5) |
|  |  |  | Category 3–4 | 4,406 | 62.3 (24.2; 81.2) |
|  |  |  | Category 5 | 59 | 13.0 (−864.4; 92.2) |
|  |  |  |  |  |  |
|  |  | Time since vaccination (years) | 0–<1 | 12,547 | 58.8 (24.4; 77.5) |
|  |  |  | 1–<2 | 8,372 | 65.3 (23.6; 84.3) |
|  |  |  | ≥2 | 4,125 | 71.9 (6.6; 91.5) |
|  | **CD** | Age group | 50–59 | 1,810 | 90.4 (29.5; 98.7) |
|  |  |  | 60–69 | 3,153 | 92.0 (41.3; 98.9) |
|  |  |  | ≥70 | 4,177 | 80.8 (52.4; 92.2) |
|  |  |  |  |  |  |
|  |  | Sex | Female | 5,159 | 83.9 (60.4; 93.5) |
|  |  |  | Male | 3,874 | 88.7 (53.4; 97.3) |
|  |  |  |  |  |  |
|  |  | Time interval between two RZV doses (days) | 28–179 | 8,185 | 88.6 (72.1; 95.3) |
|  |  |  | 180–<365 | 1,199 | 61.8 (−68.1; 91.3) |
|  |  |  |  |  |  |
|  |  | Medication use^a^ | Category 1 | 2,964 | 70.9 (4.4; 91.1) |
|  |  |  | Category 2 | 1,709 | 85.5 (−8.8; 98.1) |
|  |  |  | Category 3–4 | 4,693 | 90.6 (70.3; 97.0) |
|  |  |  | Category 5 | 7 | 100 (.; 100) |
|  |  |  |  |  |  |
|  |  | Time since vaccination (years) | 0–<1 | 9,033 | 79.1 (48.1; 91.6) |
|  |  |  | 1–<2 | 5,762 | 87.8 (49.8; 97.1) |
|  |  |  | ≥2 | 2,711 | 100 (.; 100) |
| **SLE** | | Age group | 50–59 | 1,304 | −133.9 (−667.1; 28.7)^b^ |
|  |  |  | 60–69 | 2,298 | 70.9 (18.6; 89.6) |
|  |  |  | ≥70 | 2,526 | 74.2 (35.4; 89.7) |
|  |  |  |  |  |  |
|  |  | Sex | Female | 5,386 | 53.1 (17.4; 73.4) |
|  |  |  | Male | 678 | 100 (.; 100) |
|  |  |  |  |  |  |
|  |  | Time interval between two RZV doses (days) | 28–179 | 5,373 | 56.8 (24.0; 75.5) |
|  |  |  | 180–<365 | 878 | 100 (.; 100) |
|  |  |  |  |  |  |
|  |  | Medication use^a^ | Category 1 | 4,749 | 63.2 (28.7; 81.0) |
|  |  |  | Category 2 | 1,339 | 48.1 (−51.2; 82.2) |
|  |  |  | Category 3–4 | 39 | 100 (.; 100) |
|  |  |  |  |  |  |
|  |  | Time since vaccination (years) | 0–<1 | 6,064 | 55.0 (9.0; 77.7) |
|  |  |  | 1–<2 | 3,730 | 81.9 (24.1; 95.7) |
|  |  |  | ≥2 | 1,681 | 32.1 (−146.9; 81.3) |
| **MS** | | Age group | 50–59 | 2,199 | 46.2 (−56.8; 81.5) |
|  |  |  | 60–69 | 3,385 | 18.7 (−65.1; 59.9) |
|  |  |  | ≥70 | 2,188 | 77.4 (26.3; 93.1) |
|  |  |  |  |  |  |
|  |  | Sex | Female | 5,784 | 50.9 (11.2; 72.8) |
|  |  |  | Male | 1,891 | 35.1 (−94.4; 78.3) |
|  |  |  |  |  |  |
|  |  | Time interval between two RZV doses (days) | 28–179 | 6,900 | 41.2 (−1.0; 65.8) |
|  |  |  | 180–<365 | 1,031 | 81.6 (−39.0; 97.6) |
|  |  |  |  |  |  |
|  |  | Medication use^a^ | Category 1 | 6,744 | 44.7 (5.1; 67.7) |
|  |  |  | Category 2 | 163 | 100 (.; 100) |
|  |  |  | Category 3–4 | 820 | 71.8 (−120.8; 96.4) |
|  |  |  | Category 5 | 18 | 100 (0.0; 100) |
|  |  |  |  |  |  |
|  |  | Time since vaccination (years) | 0–<1 | 7,675 | 48.6 (−9.3; 75.8) |
|  |  |  | 1–<2 | 4,670 | 62.7 (−6.3; 86.9) |
|  |  |  | ≥2 | 2,224 | 21.8 (−115.2; 71.6) |
| **PsO** | | Age group | 50–59 | 4,980 | 49.3 (−8.0; 76.2) |
|  |  |  | 60–69 | 10,622 | 82.6 (62.6; 91.9) |
|  |  |  | ≥70 | 14,358 | 80.5 (65.7; 88.9) |
|  |  |  |  |  |  |
|  |  | Sex | Female | 15,843 | 75.7 (60.6; 85.0) |
|  |  |  | Male | 13,827 | 79.5 (60.9; 89.2) |
|  |  |  |  |  |  |
|  |  | Time interval between two RZV doses (days) | 28–179 | 27,144 | 79.8 (69.2; 86.8) |
|  |  |  | 180–<365 | 3,340 | 42.1 (−51.8; 77.9) |
|  |  |  |  |  |  |
|  |  | Medication use^a^ | Category 1 | 20,836 | 76.1 (61.4; 85.3) |
|  |  |  | Category 2 | 3,967 | 90.9 (62.6; 97.8) |
|  |  |  | Category 3–4 | 3,510 | 84.6 (36.0; 96.3) |
|  |  |  | Category 5 | 2,115 | 52.2 (−14.1; 80.0) |
|  |  |  |  |  |  |
|  |  | Time since vaccination (years) | 0–<1 | 29,670 | 78.9 (64.4; 87.5) |
|  |  |  | 1–<2 | 18,576 | 81.2 (59.6; 91.3) |
|  |  |  | ≥2 | 8,703 | 55.9 (−5.8; 81.6) |
| **PsA** | | Age group | 50–59 | 1,552 | −9.2 (−323.3; 71.8)^b^ |
|  |  |  | 60–69 | 2,897 | 81.0 (38.6; 94.1) |
|  |  |  | ≥70 | 2,695 | 63.7 (14.9; 84.6) |
|  |  |  |  |  |  |
|  |  | Sex | Female | 3,814 | 60.7 (23.6; 79.8) |
|  |  |  | Male | 3,237 | 79.0 (11.4; 95.0) |
|  |  |  |  |  |  |
|  |  | Time interval between two RZV doses (days) | 28–179 | 6,375 | 63.0 (30.4; 80.3) |
|  |  |  | 180–<365 | 939 | 80.8 (−46.0; 97.5) |
|  |  |  |  |  |  |
|  |  | Medication use^a^ | Category 1 | 1,998 | 42.8 (−67.4; 80.4) |
|  |  |  | Category 2 | 809 | 80.1 (−51.3; 97.4) |
|  |  |  | Category 3–4 | 4,267 | 72.0 (35.1; 87.9) |
|  |  |  | Category 5 | 205 | 27.8 (−594.7; 92.5) |
|  |  |  |  |  |  |
|  |  | Time since vaccination (years) | 0–<1 | 7,051 | 57.6 (10.8; 79.8) |
|  |  |  | 1–<2 | 4,348 | 75.6 (20.2; 92.5) |
|  | |  | ≥2 | 1,946 | 73.8 (−106.5; 96.7) |

^a^Medication categories are detailed in **Supplementary Table 1**.

^b^The VE is not reliable due to a small number of patients.

AID, autoimmune disease; CD, Crohn’s disease; CI, confidence interval; HZ, herpes zoster; IBD, inflammatory bowel disease; MS, multiple sclerosis; N, total number of patients; PsA, psoriatic arthritis; PsO, psoriasis; RA, rheumatoid arthritis; SLE, systemic lupus erythematosus; UC, ulcerative colitis; VE, vaccine effectiveness.
